# A mixture of Nordic berries improves cognitive function, metabolic function and alters the gut microbiota in C57Bl/6J male mice

**DOI:** 10.3389/fnut.2023.1257472

**Published:** 2023-10-03

**Authors:** Fang Huang, Nittaya Marungruang, Isak Martinsson, Lluís Camprubí Ferrer, Thao Duy Nguyen, Thamani Freedom Gondo, Eva Nordberg Karlsson, Tomas Deierborg, Rickard Öste, Lovisa Heyman-Lindén

**Affiliations:** ^1^Division of Biotechnology, Department of Chemistry, Lund University, Lund, Sweden; ^2^Aventure AB, Lund, Sweden; ^3^Berry Lab AB, Lund, Sweden; ^4^Experimental Neuroinflammation Laboratory, Department of Experimental Medical Science, Lund University, Lund, Sweden; ^5^Department of Food Technology, Engineering and Nutrition, Lund University, Lund, Sweden; ^6^Department of Chemistry, Centre for Analysis and Synthesis, Lund University, Lund, Sweden; ^7^Department of Experimental Medical Science, Lund University, Lund, Sweden

**Keywords:** berries, high-fat diet, cognitive function, gut microbiota, metabolic function, neuroinflammation

## Abstract

Our diets greatly influence our health. Multiple lines of research highlight the beneficial properties of eating berries and fruits. In this study, a berry mixture of Nordic berries previously identified as having the potential to improve memory was supplemented to young C57Bl/6J male mice to investigate effects on cognition function, metabolic health, markers of neuroinflammation, and gut microbiota composition. C57Bl/6J male mice at the age of 8 weeks were given standard chow, a high-fat diet (HF, 60%E fat), or a high-fat diet supplemented with freeze-dried powder (20% dwb) of a mixture of Nordic berries and red grape juice (HF + Berry) for 18 weeks (*n* = 12 animals/diet group). The results show that supplementation with the berry mixture may have beneficial effects on spatial memory, as seen by enhanced performance in the T–maze and Barnes maze compared to the mice receiving the high-fat diet without berries. Additionally, berry intake may aid in counteracting high-fat diet induced weight gain and could influence neuroinflammatory status as suggested by the increased levels of the inflammation modifying IL-10 cytokine in hippocampal extracts from berry supplemented mice. Furthermore, the 4.5-month feeding with diet containing berries resulted in significant changes in cecal microbiota composition. Analysis of cecal bacterial 16S rRNA revealed that the chow group had significantly higher microbial diversity, as measured by the Shannon diversity index and total operational taxonomic unit richness, than the HF group. The HF diet supplemented with berries resulted in a strong trend of higher total OTU richness and significantly increased the relative abundance of *Akkermansia muciniphila*, which has been linked to protective effects on cognitive decline. In conclusion, the results of this study suggest that intake of a Nordic berry mixture is a valuable strategy for maintaining and improving cognitive function, to be further evaluated in clinical trials.

## 1. Introduction

In 2017, 11 million deaths and 255 million disability-adjusted life-years (DALYs) were attributable to dietary risk factors, among which low intake of fruit was one of the leading dietary risk factors for deaths and DALYs globally and in many countries (1, 2). The inclusion of fruits into the diet is linked to positive effects on health. It is a suggested strategy for preventing public health issues such as obesity (3), cardiovascular diseases, and cognitive dysfunction (4–6). As life expectancy increases, the global population is aging, increasing the risk for dementia and vascular diseases. In the US, the proportion of the population aged 65 years or older is projected to increase to one in five adults by 2030 (7).

The globalization and modernization of food have had a profound health impact on people’s diets worldwide (8, 9). Previous studies have shown that people are shifting away from diets high in complex carbohydrates and fiber toward more varied diets with a higher proportion of fats, saturated fats, and sugar (10, 11). This shift in dietary patterns, often associated with increased high-fat intake, has been linked to adverse health effects, including cognitive impairment (12). Given the potential consequences of cognitive decline, including Alzheimer’s disease (AD), it is essential to investigate interventions promoting healthy aging, specifically focusing on brain health. Implementing dietary interventions that positively impact the brain, cognitive function, and cardiometabolic outcomes is crucial in combating the cognitive impairment associated with global aging and unhealthy dietary patterns characterized by high fat intake.

Fruits are a vital part of a balanced diet. Among them, berries are particularly promising due to their rich content of potentially bioactive compounds, including polyphenols and fiber. These colorful fruits have gained increasing attention for their health merits and lately their beneficial effects on brain function (13–16). Several different types of berries have been associated with improved cognitive performance, including commonly consumed berries like American highbush blueberries (*Vaccinium ashei, Vaccinium virgatum, or Vaccinium corymbosum*) (17–20), lowbush wild blueberries (*Vaccinium angustifolium*) (21–23), raspberries (24), strawberries (25, 26), and red grapes (27–29), etc. There is high variation amongst different types of berries in the quantity and composition of compounds belonging to different polyphenolic classes, as well as other nutrients such as fiber. Hence, different berry species could possess some common health effects, but likely also unique health properties reflecting the specific profile of bioactive compounds present in that particular berry. Bioactive compound content is also greatly affected by environmental conditions.

Nordic environments are rich in a variety of wild berries that grow abundantly in for example Scandinavian forests. Several of these berries have been shown to be rich in phenolic compounds and dietary fibers (30). Anthocyanins, which are regarded as important for the health properties of berries on human metabolism (31, 32), have also been shown to increase in bilberries growing in northern latitudes (33) as compared to southern parts of Europe (34). Nonetheless, several of these Nordic berries remain largely unexplored for their potential health benefits, especially in relation to memory and cognitive function. There are studies on lingonberry (*Vaccinium vitis-idea*) and bilberry (aka European blueberry, *Vaccinium myrtillus*) which show beneficial effects associated with reduced inflammation (35, 36), reduced blood pressure (37) as well as improvement of brain function in mouse models (38, 39). In our recent study including a screening of lesser-known wild berries from the Nordic countries, we found for the first time that several Nordic berry species mediate positive effects on spatial memory in middle-aged mice (39). However, cognitive outcomes and underlying mechanisms by the berries of interest remain to be further elucidated.

In preclinical studies, several groups have found that a high-fat diet can lead to cognitive impairment in rodents, including deficits in memory and learning; these deficits are thought to be related to changes in the brain’s structure and function, as well as to increased neuroinflammation level (40–42).

The gut microbiota has been demonstrably linked to multiple dimensions of human health. This includes its role in preventing pathogen colonization (43), supporting the function of the intestinal epithelium (44), metabolizing both dietary and pharmaceutical compounds (44, 45), and modulating immune function (46). Beyond these roles, the gut microbiota can also influence neuroinflammation (47), behavior and cognitive function (47–49). Specifically, gut microbes can produce signaling molecules, including neurotransmitters and hormones, which may affect brain function and behavior (i.e., the gut-brain axis). Moreover, ingested food reaching the gut will be fermented by certain bacteria to produce short-chain fatty acids (SCFAs). These SCFAs not only serve as a byproduct but also promote the proliferation of beneficial bacteria within the gut (50). Hence, our diet and especially foods rich in dietary fiber are essential in shaping the gut microbiota composition and function. Berry intake is shown to influence the signal pathways involved in microbiota-brain axis and exert beneficial effects on cognitive function and inflammation (48, 51, 52).

The present study aims to assess the effects of a Nordic berry mixture containing lingonberries and bilberries on cognition and memory in young mice. In addition, plausible mechanisms were addressed that may mediate the beneficial effects of supplementation with these particular Nordic berries. For this purpose, markers of neuroinflammation, synaptic function, and neurogenesis were assessed in the hippocampus, a brain region involved in spatial learning and memory (53). Additionally, the effects of berry supplementation on systemic markers of cardiometabolic function and the composition of the gut microbiota were assessed.

## 2. Materials and methods

### 2.1. Experimental diets

The berry mixture was developed as a study product in a clinical trial (NCT04317612) to produce a drinkable product with a reasonably palatable taste containing berries with potentially different health merits. It contains lingonberries, bilberries, and grape juice. The proprietary blend contained all parts of the berries and was stored protected from light and oxygen until freeze-dried at SLU Balsgård (Kristianstad, Sweden). The resulting berry mixture powder was flushed with nitrogen gas, vacuum-packed in bags with oxygen absorbers, and stored at room temperature until incorporated into rodent diet pellets [Research Diets, Inc. (New Brunswick, NJ, USA)].

The diets were a high-fat (HF) control diet (60% calories from fat, modified from D12492) and a HF diet supplemented with berry mixture powder (HF + Berry) at a dose of 20% (w/w), dry weight basis. The diets were formulated to be matched on macro- and micronutrients (Supplementary Tables 1, 2). The diets were stored in bags flushed with nitrogen gas and stored at −20°C until use. A standard chow diet (RM1, SDS) was also included in the study as a control group. High-fat diets are commonly used in research to investigate the effects of dietary interventions on the development of obesity and associated disorders, which have been linked to impaired cognitive function (54–56).

### 2.2. Quantification of carbohydrate composition of the berry mixture powder

A two-step sequential acid hydrolysis process was carried out to quantify the content of carbohydrates in the various berry fibers, essentially as described previously by Zambrano et al. (57). The monosaccharide contents of the freeze-dried berry mixture sample were analyzed by High-Performance Anion Exchange Chromatography (HPAEC) (Thermo Fisher Scientific, Waltham, USA). The separation of the monosaccharides was carried out using a Dionex CarboPac PA-20 analytical column coupled to a Dionex CarboPac PA-20 guard column of the same material and detected by a Pulsed Amperometric Detector. The standards employed in this method included arabinose, xylose, glucose, galactose, mannose, and fructose. The results were based on triplicates measurements and the values were shown as mean and standard deviation (SD).

### 2.3. Polyphenol analysis of the berry mixture powder

The polyphenols analysis method was executed following a modified proposal from Marzullo et al. (58). Briefly, 1 gram of the freeze-dried berry powder, underwent extraction using a 10 mL solvent solution comprising 1% formic acid in a 50/50 v/v mixture of methanol and water. The analysis was conducted utilizing an Agilent HPLC 1,100 series system equipped with a diode array detector (DAD) (Agilent Technologies, Waldbronn, Germany) and Kromasil RP-C-18 column (150 × 4.6 mm, 3.5 μm). Total phenolics, flavonoids, and anthocyanins were quantified at 280, 360, and 520 nm, respectively. Quantification of total phenolics was performed relative to gallic acid standard, whereas flavonoids were quantified relative to quercetin standard. Lastly, anthocyanins were quantified using cyanidin 3-O glucoside equivalence.

### 2.4. Animal experiment

Thirty-six male C57Bl/6J mice (Janvier-Labs, Le Genest-Saint-Isle, France) arrived at the animal facility at the age of 5 weeks and were given standard chow (RM1, SDS) *ad libitum* for 3.5 weeks for acclimatization (22°C, 12 h light-dark cycle). Subsequently, the mice were allocated into weight-matched groups of 12 animals/diet and randomly assigned to receive one of the three diets (HF, HF + Berry, or chow) for 4.5 months.

Animals and food were weighed, and the food was replaced every week. The food intake per cage was measured weekly based on three cages (*n* = 4 animals/cage) per diet group. The calorie intake was calculated and presented as the average consumption per mouse. The study was approved by the local animal experiment ethical review committee in Lund, Sweden (approval number 5.8.18-13983/2018).

### 2.5. Behavior

Behavior tests were performed in a separate room dedicated to only this purpose. Light density in the room was adjusted and kept at 45 lux. All behavioral tests were conducted once, and every animal within each respective group underwent all of the tests, resulting in a total of 12 animals tested per diet group. The T-maze spontaneous alternation test and novel object recognition (NOR) test were conducted after 13–14 weeks after the mice were put on the research diets. Barnes maze was conducted in the feeding week of 16–18. For all behavioral tests, monitoring was performed with a camera system using the Ethovision XT 14.0 software (Noldus Information Technology b.v., Wageningen, Netherlands) for both video tracking and video analysis.

#### 2.5.1. T-maze

The T-maze was made of transparent acrylic and consisted of three arms (two goal arms: 30 cm × 10 cm; one starting arm: 30 cm × 10 cm). In this study, a discrete-trial-without-a-reward protocol was adapted from a previous Y-maze protocol (39, 59). The protocol was based on the tendency of animals to explore the novel arm rather than the one previously explored without rewarding stimuli. The correct first turn (%) was calculated as the percentage of the animals that made a correct turn at their first choice of turning; frequency (%) was calculated as the visiting frequency to the correct arm in ratio to the total visits in both arms; duration (%) was calculated as the time spent (seconds) in the correct arm in ratio to the total time spent (second) in both arms.

#### 2.5.2. Novel object recognition (NOR) test

A modified novel object recognition (NOR) protocol was followed as previously described (39) with minor modifications. The objects used in the current study were either glass balls (diameter 2.5 cm) or acrylic dice (2.5 cm^3^). In the test session, the visits to and the time spent on exploring the novel object in ratio to the total visits to and the total time spent on both objects were calculated–frequency (%) and duration (%), respectively–to reveal the animal’s preference for the novel object. The term “Position B” was employed in the video analysis and referred to the position where the novel object was placed, considering that the novel object position was randomly chosen between one of the two positions to avoid any orientation preference of the animals.

#### 2.5.3. Barnes maze

**FIGURE 1:**
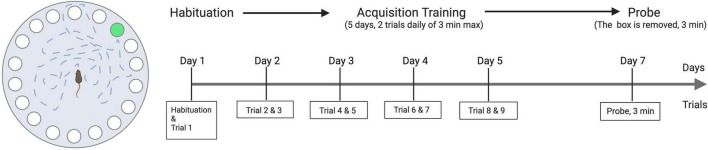
Illustration of Barnes maze apparatus and the experimental scheme.

The Barnes maze protocol previously described by Attar was adapted to the study (60). The apparatus used (Noldus Information Technology b.v., Wageningen, The Netherlands) is a 100-cm diameter table with 20 holes lining the perimeter. One of the holes has a box underneath it that mice can use to escape the table. The Barnes maze test included 11 trials for each animal: one habituation trial, nine training trials, and one probe trial, the Barnes maze test was conducted once per animal. The first day of the test started with a habituation trial where the experimenter used an opaque cylinder to transfer the animal to the center of the table; the animals then spent a maximum of 3 min learning about the existence of the hidden box and recognized it as a place to hide in Figure 1. The habituation trail was then followed 1 h later by the first training trial. During the training trials, the animals were again given a maximum of 3 min to find the box. Each mouse performed two training trials per day for the following four consecutive days to learn the location of the escape hole. Two days after the last training trial, a single “probe trial” was performed where no box was placed beneath the target hole. The test animal stayed on the table for 3 min. During the training trials, the time to go to the box was recorded for each animal. During the probe trial, the latency to the target hole, the average distance of the animal to the target hole, and the time spent in the target quadrant were measured.

### 2.6. Sample collection

At the end of the study, the animals were fasted for 4 h before being anesthetized using isoflurane (Abbott, Chicago, IL, USA). A cardiac puncture was carried out for blood sampling before the mice were terminated. Immediately after termination, the brains and other tissues were dissected, and the blood plasma samples were separated by centrifugation at 2,000 × *g* for 10 min. The cecum was dissected from each animal using sterile instruments and was preserved in DNA-free tubes. The cecum content was used in the analysis of gut microbiota.

All collected tissues were snap-frozen and subsequently stored at −80°C until further analyses.

### 2.7. Brain immunohistochemistry

The right hemispheres of mice were dissected, fixed in paraformaldehyde for 72 h, and embedded in a cryomount for cryosectioning. A total of 16 sections (12 μm) per animal (a total of 320 sections covering 192 μm of the hippocampal dental gyrus) were cryosectioned from 10 animals in each group (the HF group and the HF + Berry group). The sections were treated in several steps to prepare for immunolabelling, including heat-induced epitope retrieval (61), peroxidase quenching, blocking, and application of primary anti-doublecortin (DCX) antibodies followed by horseradish peroxidase (HRP)-conjugated secondary antibodies. Visualization of binding sites was performed using 3,3’-diaminobenzidine/H_2_O_2_ reaction, and nuclei were stained with Mayers hematoxylin. The sections were dehydrated, cleared in xylene, and mounted with Pertex mounting media. The neuroanatomically defined dentate gyrus was annotated. Both manual and digital (ImageJ-based macro) methods were used to quantify the number of labeled cell bodies and the area of labeled dendrites.

### 2.8. Hippocampal homogenates

Hippocampi from left hemispheres (*n* = 12 per group) were dissected and stored at −80°C for biochemistry analyses. Using an electric homogenizer, the tissues were homogenized on ice in Tris-buffered saline with a 1% triton-X100 and 1% protease inhibitor cocktail. The methodology refers to as described previously (39).

### 2.9. Quantification of hippocampal cytokines and brain-derived neurotrophic-factor

Cytokine levels were measured in hippocampal homogenates using Meso Scale Discovery (MSD) V-PLEX proinflammatory panel one kit (#K15048D-1, Meso Scale Diagnostics, Rockville, MD, USA), a method based on electrochemiluminescence. Briefly, biological reagents bind carbon electrodes within the multi-spot wells. Electrochemiluminescent labels that are conjugated to the detection antibodies (SULFO-TAG) are used to detect the analytes allowing for ultrasensitive detection. Electricity is then applied to the electrodes through the MSD instrument leading to light emission by the SULFO-TAG labels and the intensity of the light is measured to quantify the biological analytes in the sample. The kit includes the following analytes, and their calculated detection limits are stated in parenthesis: IFN-γ (0.0402–909 pg/mL), IL-1β (0.569–1765 pg/mL), IL-2 (0.153–2,595 pg/mL), IL-4 (0.0707–1,680 pg/mL), IL-5 (0.308–1,027 pg/mL), IL-6 (0.674–5,440 pg/mL), IL-10 (0.161–3,930 pg/mL), IL-12p70 (2.72–31,400 pg/mL), KC/GRO (0.195–1,960 pg/mL), TNF-α (0.0631–619 pg/mL). Statistical analysis was performed only in the cytokines consistently appearing within detection limits throughout the groups. Plates were read using a QuickPlex Q120 reader (Meso Scale Diagnostics, Rockville, MD, USA). Total protein in hippocampal homogenates (1:1 dilution) was determined by bicinchoninic acid (BCA) assay and used to normalize protein levels prior to loading in the mesoscale plates. Analysis was performed by comparing the detected light intensities and comparing them to a calibration sample of known quantities.

Brain-derived neurotrophic factor (BDNF) (Human BDNF SimpleStep ELISA^®^ kit, Abcam PLC, Cambridge, UK) and nerve growth factor (NgF) (Mouse NgF ELISA^®^ kit, MyBioSource, Inc., San Diego, CA, USA) in hippocampus homogenates were determined by enzyme-linked immunosorbent assays. BDNF results were reported per mg protein in the brain, and the brain homogenates were made on different weights of the hippocampus. The method involves specific antibodies selectively binding to the target protein such as BDNF, followed by an enzymatic reaction that produces a color change. The intensity of this color change allows for the quantification of the target protein in the samples. All experiments were conducted in accordance with the manufacturer’s protocol.

### 2.10. Western blot analysis of hippocampal synaptic markers

To detect hippocampal memory-related proteins, the sample homogenates were mixed with Novex™ Tricine SDS Sample Buffer (LC1676, Invitrogen™, Thermo Fisher Scientific Inc., Sweden), heated at 85°C for 2 min, shortly centrifuged and with equal amounts of protein loaded onto a Novex™ 10 to 20% Tricine gel (EC6625BOX, Novex™, Thermo Fisher Scientific Inc., Sweden) and run at 90 v for 3 h at room temperature. After SDS PAGE gel electrophoresis, the proteins were transferred to a PVDF membrane using the iBlot™ 2 machine (Thermo Fisher Scientific Inc., Sweden). All membranes were blocked in phosphate-buffered saline with Tween™ 20 (PBS-T) containing 5% BSA (Thermo Fisher Scientific Inc., Sweden). The membranes were incubated overnight with primary antibodies at 4°C, followed by secondary horseradish peroxidase (HRP)-conjugated antibodies for 1 h at room temperature. All washes were done in PBS-T. The membrane was developed using enhanced chemiluminescence (ECL) Substrate and visualized using a BioRad Chemidoc XRS + system (Bio-Rad, Hercules, CA, USA). Quantification of the bands was performed using Image Lab 6.1. All bands were normalized to Tubulin, and values were given as fold changes to the HF diet.

The antibodies employed in this method were the following: glutamate A1 (GluA1) (Merck Millipore, Sweden), anti-N-methyl-D-aspartate receptor (NR1) (Upstate^®^, New York, NY, USA), post-synaptic density-95 (PSD-95) (Merck Millipore, Sweden), anti-synaptophysin (Merck Millipore, Sweden), anti-Alpha Tubulin (Sigma-Aldrich, Sweden), anti-cAMP response element binding protein (CREB)-[LB9] (Abcam, Cambridge, UK), anti-phospho CREB Ser133 [E113] (Abcam, Cambridge, UK), anti-FBJ murine osteosarcoma viral oncogene homolog B (FOSB) [5G4] (Cell Signaling Technology, Inc., Sweden), HRP-conjugated secondary antibodies (Biotechne, UK).

### 2.11. Plasma levels of metabolic and inflammatory markers

Fasting glucose, total, and high-density lipoprotein (HDL) cholesterol levels were measured in plasma using Infinity™ Glucose Hexokinase Liquid Stable Reagent, Infinity™ Cholesterol, and HDL-CHOLESTEROL Plus Direct method, respectively (Thermo Fisher Scientific Inc., Waltham, MA, USA). Insulin levels were quantified by enzyme immunoassay kits (Mercodia AB, Uppsala, Sweden). Lipopolysaccharide binding protein (LBP) was measured by mouse LBP ELSA kit (Hycult Biotech Inc., Wayne, NJ, USA). Serum amyloid A (SAA) levels were determined by “PHASE” ™ Murine Serum Amyloid A Assay (Tridelta Development Ltd., Maynooth, Ireland). Cytokine levels were measured in plasma (dilution 1:1) using Meso Scale Discovery V-PLEX proinflammatory panel one kit (#K15048D-1) as described in section 2.8. In this case, the calculated detection limits were the following: IFN-γ (0.00988–909 pg/mL), IL-1β (0.374–1,765 pg/mL), IL-2 (0.194–2,595 pg/mL), IL-4 (0.0266–1,680 pg/mL), IL-5 (0.224–1,027 pg/mL), IL-6 (0.335–5,440 pg/mL), IL-10 (0.103–3,930 pg/mL), IL-12p70 (1.79–31,400 pg/mL), KC/GRO (0.0968–1,960 pg/mL), TNF-α (0.0256–619 pg/mL).

### 2.12. Cecal microbiota

Mouse cecal samples (12 per group) were shipped to Clinical Microbiomics Lab (Copenhagen, Denmark) for 16S DNA sequencing according to the standard routines performed at the lab. Briefly, DNA of 88 mouse cecal samples were successfully extracted using NucleoSpin^®^ 96 Soil (Macherey-Nagel) with additional bead-beating steps. The V3-V4 region of the 16S rRNA genes were amplified and sequenced on an Illumina MiSeq desktop sequencer using the MiSeq Reagent Kit V3 (Illumina) for 2 × 300 bp paired-end sequencing. Data obtained from an average sequencing depth of 28,194 read pairs per sample after quality filtering (Illumina MiSeq 300PE) were used for bioinformatics analysis. An adjusted dada2 pipeline was used to process the sequence data into an amplicon sequence variant abundance table (62). The default taxonomic assignment of the detected ASVs was done using a naïve Bayesian classifier algorithm comparing the ASV sequences to the SILVA reference database (v138.1).

### 2.13. Statistical analyses

Data are presented as mean ± SD and analyzed by one-way ANOVA followed by Dunnett’s test for multiple comparisons versus the HF control group. The graphs presenting Barnes maze and body weight result display mean ± SEM. In T-maze data, one sample t-test and Wilcoxon test were applied to compare each group’s % frequency and % duration against 50% chance. Non-parametric Wilcoxon signed ranks test was used in NOR data. DCX quantitation data in brain immunohistology was assessed with t-test and Mann–Whitney U tests. The ROUT method was applied to identify outliers for each biomarker aiming at the maximum desired false discovery rate (Q) of 5.

For the 16S data, the diversities analyzed by the Shannon index from each group were compared to the HF control group using the Kruskal–Wallis rank sum test, followed by pairwise comparisons using the Wilcoxon rank sum test. The Unique observed species and Total OTU richness were compared using the ANOVA test followed by the least significant difference (LSD) *post hoc* test when ANOVA indicated significance. The OTU data from each group were compared to the HF control group using two-way ANOVA, and *p*-values were corrected for multiple comparisons by controlling the False Discovery Rate (FDR) using the original FDR method of Benjamini and Hochberg. If the data were normally distributed, data would be presented as means in bar charts; otherwise, the data would be presented as median values in box plots.

GraphPad Prism version 8.01 (GraphPad Software, Inc., La Jolla, CA, USA) was used for statistical analyses.

## 3. Results

### 3.1. Berry powder composition and diet intake, body weight, organ weight

The nutrient analysis showed that the freeze-dried berry mixture had 13.7% (SD = 2.7) dietary fiber and 68.0% (4.8) carbohydrates; the rest were fat (3.5%, 0.18), protein (4.0%, 0.2), and moisture content (9.2%, 0.09). Moreover, the analysis of carbohydrates composition further revealed 24.0% (4.8) fructose and 23.2% (4.6) glucose as free sugars in the berry mixture powder, and the hydrolyzed glucose (48.3%, 1.1) was higher than the free glucose. In addition, 1.9% (0.1) of xylose, 1.8% (0.1) of arabinose, and 1.2% (0.1) of galactose were determined in the carbohydrate compositions of the berry powder (Supplementary Table 1). The analysis of carbohydrates composition in this study suggests that the dietary fiber in the berry mixture powder contained the hemicellulose xyloglucan, as well as components that are most likely neutral side chains from pectic polysaccharides [e.g., (arabinan, arabinose and galactose)].

The freeze-dried berry powder was added to the final diet composition at a concentration of 20% (w/w), as presented in Supplementary Table 2.

**FIGURE 2:**
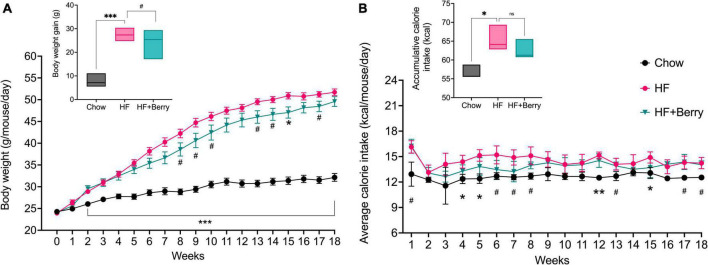
The effect of diets on body weight and calorie intake; **(A)** The graph presents the weekly body weight of the animals and the final body weight gain in shown in the inset; **(B)** The graph depicts the average calorie intake per mouse in the different diet groups, measured per cage (3 cages per diet group) and the inset shows the total accumulative calorie intake per mouse. Symbols indicate black dot–the chow group; pink dot–the HF group; green triangle–the HF + Berry group. Two-way and One-way ANOVA were used for data analyses, and Dunnett’s multiple comparisons test was used for *post hoc* analysis to compare each diet to the HF diet. No significant difference or trend (ns). Significant differences denoted by *p < 0.05, **p < 0.01, and ***p < 0.001. (^#^*p* < 0.1 denotes a non-significant trend). Values are represented as mean ± SEM (Body weight) and mean ± SD (calorie intake) for *n* = 12 per diet group.

At the beginning of the feeding period, there was no significant difference in mean body weight among the three groups: chow (24.3 g, SD = 1.6), HF (23.9 g, SD = 1.3), and HF + Berry (24.1 g, SD = 1.7) (Figure 2). By the end of the study, the mean body weight of the chow group was 32.2 g (SD = 3.1), the HF group 51.7 g (SD = 2.6), and the HF + Berry group weighed 49.6 g (SD = 3.9). After 4.5 months on diets, the body weight gain was significantly higher in the mice receiving the HF diet compared to the chow diet (Figure 2). There was an overall tendency for the mice receiving the HF diet supplemented with the berry mixture to gain less body weight compared to the HF control group (*p* = 0.06), with trends of lower body weight in weeks 8, 9, 10, 13, 14, and 17 (*p* < 0.1) and a significantly reduced body weight compared to the HF group at week 15 (*p* < 0.05) (Figure 2A). The mice receiving the HF diet consumed significantly (*p* < 0.05) more energy compared to the chow group in weeks 4, 5, 12 (*p* < 0.01), and 15; and by the end of the feeding period, the HF group had significantly higher (*p* < 0.05) total accumulative calorie intake (Figure 2B). There were no significant differences in calorie intake between the HF and HF + Berry groups (Figure 2B).

The HF group showed significantly higher weights of livers (mean ± SD: 2.6 ± 0.3 g, *p* < 0.001) and epididymal fat pads (1.3 ± 0.2 g, *p* < 0.001) compared to the chow group (liver 1.4 ± 0.2 g, fat 0.6 ± 0.2 g). Mice receiving the high-fat diet supplemented with berries had significantly lower liver weights (2.2 ± 0.5 g, *p* < 0.05) compared to the group receiving the HF without berries (2.6 ± 0.3 g). The HF + Berry group had larger epididymal fat (1.6 ± 0.3 g, *p* < 0.01) compared to the HF group (1.3 ± 0.2 g). Brain weight did not differ between the groups (data not shown).

### 3.2. Effects on memory performance in T-maze and NOR tests

**FIGURE 3:**
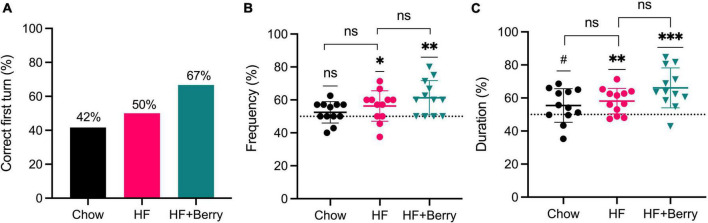
Effects of the chow diet, the HF diet, and the HF + Berry diet on the T-maze test; **(A)** Correct first turn (%) calculated as the percentage of the animals that have made a correct turn at their first choice of turning; **(B)** Frequency (%) calculated as the visiting frequency to the correct arm in ratio to the total visits in both arms; **(C)** Duration (%) calculated as the time spent (second) in the correct arm in ratio to the total time spent (second) in both arms. The data was extracted after the animals spent 1 min of exploration in the choice run. Symbols indicate black dot–the chow group; pink dot–the HF group; green triangle–the HF + Berry group. Wilcoxon signed-rank test was applied to compare each group against a 50% chance; one-way ANOVA and Dunnett’s multiple comparisons test was used for *post hoc* analysis to compare each diet to the HF diet. Significant differences denoted by *p < 0.05, **p < 0.01, and ***p < 0.001. (^#^*p* < 0.1 denotes a non-significant trend). Values are represented as mean ± SD for *n* = 12 per diet group.

In the group fed the HF diet with the berry mixture, 67% of the animals made the correct choice on their first turn, as shown in Figure 3A. In the chow and HF groups, 42 and 50%, respectively, made the correct choice (Figure 3A). The berry group visited the unexplored arm more often (Figure 3B) and spent more time in the correct arm (Figure 3C), compared to random chance (**p < 0.01 and ***p < 0.001, respectively). The group fed the high-fat diet also showed significantly higher frequency (%) and duration (%) than 50% of random chance (*p < 0.05 and **p < 0.01, respectively). The group fed the chow did not show a significantly higher frequency (%), but a non-significant trend was observed for increased duration of time spent in the unexplored arm (Figure 3C) compared to 50% random chance. When comparing to each diet group to the HF diet, no significant difference was observed. Additionally, results from the novel object recognition test showed that all the groups spent more time exploring the new object compared to the familiar one in the test session, but there were no significant differences between the diet groups in their level of exploration on the novel object (data not shown).

### 3.3. HF + Berry group shows improved memory in Barnes maze

**FIGURE 4:**
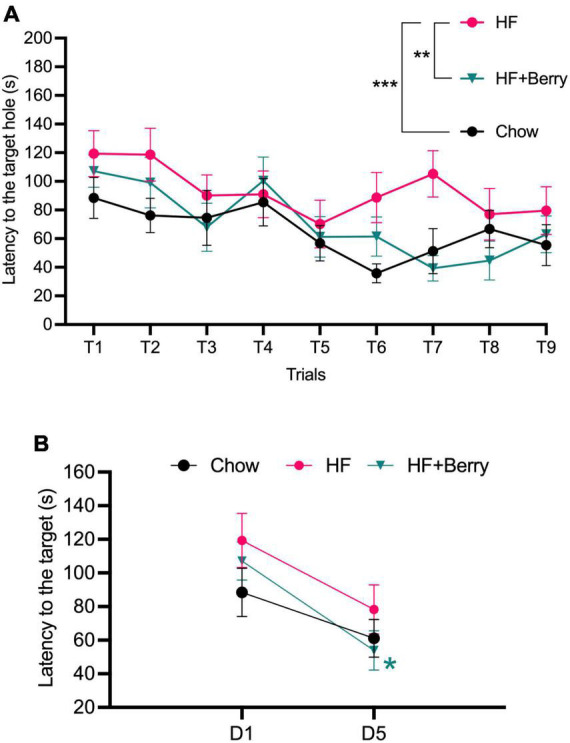
Barnes maze performance of the chow–, the HF– and the HF + Berry group. **(A)** The latency (s) until the mice entered the escape box during the training trails; **(B)** The latency of day 5 (D5) vs. day 1 (D1). Symbols indicate: black dot–the chow group; pink dot–the HF group; green triangle–the HF + Berry group. Two-way ANOVA and One-way ANOVA were applied to compare between the diets’ groups. Significant differences denoted *p < 0.05, **p < 0.01, and ***p < 0.001. Values are represented as mean ± SEM for *n* = 12 per diet group.

All groups improved in their ability to locate the target hole over the experimental period, as shown by the decreasing time needed to find the hole with an increasing number of training trials (Figure 4A). Two-way ANOVA analysis revealed that both the diet and the trial number significantly affected latency to the target hole; the HF + Berry group and the chow group performed significantly better than the HF group (**p < 0.01, ***p < 0.001, respectively). Additionally, the group on the high-fat diet supplemented with the berry mixture had significant learning improvement from the first to the last day (D1 vs. D5) of testing (Figure 4B).

During the probe trial, there were no significant differences in latency or average distance between the groups during the 3 min of exploration on the table (data not shown). All groups spent more time in the quadrant where the target hole was located compared to the other three quadrants, but there were no significant differences between the groups in this regard (data not shown).

### 3.4. Effects of berries on hippocampal synaptic function and expression of neurotrophic proteins

Since the berry mixture was found to enhance spatial memory, and the hippocampus is known to play a key role in memory and spatial navigation, we further investigated the proliferation and functionality of hippocampal neurons. Regarding synaptic proteins in the hippocampus, several differences between the diet groups were identified (Supplementary Figure 1). The mice that were fed the chow diet showed significantly higher levels of GluA1 and synaptophysin (*p* < 0.05 and *p* < 0.001, respectively) compared to those that were fed the HF diet. There were no significant differences in these two markers between the HF and HF + Berry groups (Supplementary Figures 1A, E). Although there was no significant difference in GluA1 levels between the HF and HF + Berry groups, the phosphorylated form of GluA1 (pGluA1) was significantly higher in the HF + Berry group than in the HF group (Supplementary Figure 1B). The post-synaptic protein PSD95 had a significantly higher (*p* < 0.001) level in the HF group compared to the HF + Berry group, but there was no significant difference between the HF and chow groups (Supplementary Figure 1F). There were no significant changes in protein levels for FOSB, CREB, pCREB, or NR1 (Supplementary Figures 1C, D, G, H).

There was no difference in the total protein levels of BDNF or Ngf in the hippocampus region of mice from different diet groups (data not shown).

Quantification of a neurogenesis marker–DCX–using immunolabelling the dentate gyrus did not show any significant difference between the HF and HF + Berry groups (data not shown).

### 3.5. Berries normalize anti-inflammatory IL-10 levels in hippocampus when added to high fat diet

**FIGURE 5:**
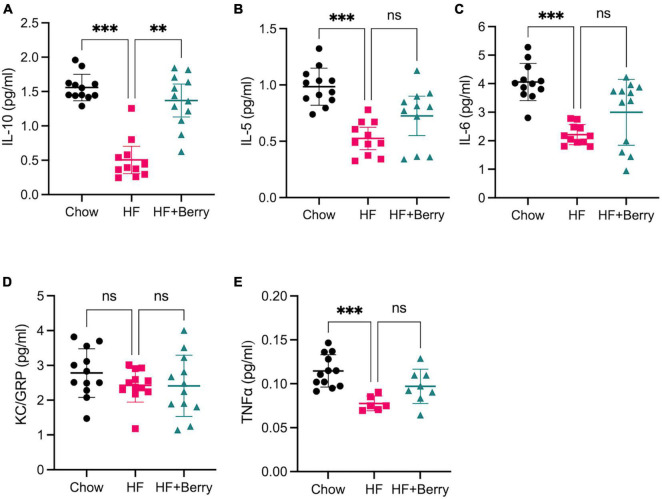
The effect of chow, HF and HF + Berry diets on cytokine levels in the hippocampus. Mesoscale results are given in pg/ml for all cytokines. **(A)** IL-10; **(B)** IL-5; **(C)** IL-6; **(D)** KC/GRO; **(E)** TNFα. Note that while TNFα levels was significantly lower in the HF group (*p* < 0.001) compared to the chow group, 6 samples from the HF group dataset were excluded as they were below the detection limit. Symbols indicate: black dot–the chow group pink dot–the HF group; green triangle–the HF + Berry group. Non-parametric Kruskal-Wallis test followed by Dunn’s test, **p < 0.01 and ***p < 0.0001. Values are represented as mean ± SD for *n* = 6–12 per diet group.

We further investigated whether the HF + Berry diet led to changes in neuroinflammation (Figure 5). The anti-inflammatory cytokine IL-10 levels in the hippocampus homogenates of mice fed the HF diet were significantly lower than those fed the chow (*p* < 0.001) and the HF + Berry (*p* < 0.01) diets (Figure 5A). Moreover, the HF group showed significantly lower (*p* < 0.001) levels of IL-5 (Figure 5B) and IL-6 (Figure 5C) compared to the chow group. Keratinocyte chemoattractant (KC)/growth-regulated oncogene (GRO) did not differ between the groups. However, TNFα levels were significantly lower in the HF group (*p* < 0.001) compared to the chow group (Figure 5E). It is worth noting that the levels of IFNγ and IL-2 were below the detection limit in the majority of the hippocampal homogenates, and therefore, the data was not presented.

### 3.6. Effect on glucose homeostasis, lipid metabolism, and markers of inflammation

**FIGURE 6:**
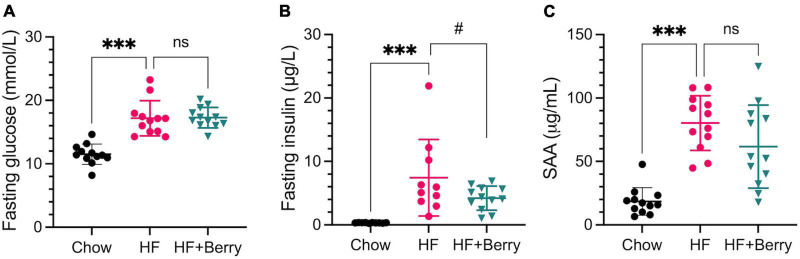
Blood plasma levels of **(A)** fasting glucose (mmol/L); **(B)** fasting insulin (μg/L); **(C)** SAA (μg/mL) in the chow–, the HF– and the HF + Berry groups. One-way ANOVA followed by Dunnett’s test was applied for multiple comparisons versus the HF control group. Significant differences denoted ***p < 0.001. (^#^*p* < 0.1 denotes a non-significant trend). Values are represented as mean ± SD for *n* = 10–12 per diet group.

The HF group showed significantly higher levels of fasting glucose, insulin and SAA compared to the chow group (Figures 6A–C), whereas there were no significant differences between the HF + Berry group and the HF group with respect to these parameters. There was a strong trend (*p* = 0.08) toward reduced insulin levels amongst the mice receiving supplementation with berries, as compared to mice receiving HF without berries.

Moreover, the HF group had significantly higher levels (*p* < 0.001) of total cholesterol, HDL cholesterol, and LBP compared to the chow group, whereas there were no differences in these parameters between the HF and the HF + Berry group (Supplementary Figures 2, 3).

The effect of HF + Berry diet on inflammation was further investigated by analyzing plasma cytokine levels (Supplementary Figure 4). Plasma levels of interferon-gamma (IFNγ), IL-10, IL-5, and IL-6 were not different between the groups (Supplementary Figures 4A, B, D, E). The HF group showed a statistically significant increase (*p* < 0.05) in IL-2 levels compared to the chow group, but no significant difference was observed between the HF + Berry group and the HF group. The levels of KC/GRO and TNFα were also significantly higher (*p* < 0.001 and *p* < 0.01, respectively) in the HF group compared to the chow group, although no significant difference was found between the HF + Berry group and the HF group (Supplementary Figures 4F, G).

### 3.7. Gut microbiota

**FIGURE 7:**
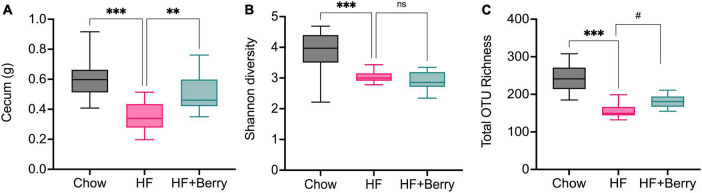
**(A)** The cecum weight and the Alpha diversity of the gut microbiota in mice fed chow diets, high-fat diets and high-fat supplemented with berry mixture diets measured by **(B)** Shannon diversity index; **(C)** Total OTU richness. The Shannon index compared each group to HF control group using Kruskal–Wallis rank sum test followed by pairwise comparisons using Wilcoxon rank sum test. Significant differences denoted **p < 0.05, ***p < 0.001. (^#^*p* < 0.1 denotes a non-significant trend). Values are represented as mean ± SD for *n* = 12 per diet group.

The cecum is a key location for microbial fermentation in mice, and in this study, we found that cecum weights were significantly higher in mice on the chow diet and the HF + Berry diet compared to the HF diet (Figure 7A). Analysis of cecal bacteria revealed that the mice on the chow diet had significantly higher microbial diversity (*p* < 0.001) as measured by the Shannon diversity index and a significantly greater (*p* < 0.001) total number of different types of bacteria as measured by the total OTU richness, compared to the HF group (Figures 7B, C). The HF + Berry group tended to have higher total OTU richness than the HF group, but there were no significant differences between the two groups in terms of the Shannon diversity index (Figures 7B, C).

**FIGURE 8:**
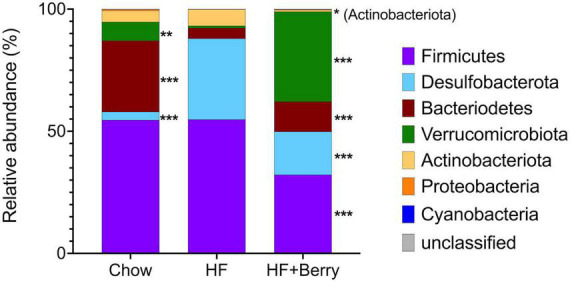
Composition of the gut microbiota at phylum level in cecum of mice fed chow, HF or HF + Berry diets for 18 weeks (*n* = 12 per group). Each group was compared to the HF control group using two-way ANOVA and the *p*-values were corrected for multiple comparisons by controlling the False Discovery Rate (FDR) using the original FDR method of Benjamini and Hochberg. Significant differences denoted *p < 0.05, **p < 0.01, and ***p < 0.001.

At the phylum level, the result showed that the mice fed the high-fat (HF) diet had a dominant population of Firmicutes (55%) and Desulfobacterota (33%), along with smaller populations of Bacteroidetes (4%), Verrucomicrobia (1%), and Actinobacteriota (7%) (Figure 8). The mice on the chow diet had similar levels of Firmicutes (55%) and Actinobacteria (5%) as the HF group but significantly lower levels of Desulfobacterota (3%, *p* < 0.001) and higher levels of Bacteroidetes (29%, *p* < 0.001) and Verrucomicrobia (8%, *p* < 0.01) compared to the HF group (Figure 8). The HF + Berry group had the highest relative abundance of Verrucomicrobiota at 37%, which was significantly higher than the chow and the macronutrient-matched HF group (both *p* < 0.001). When compared to the HF group, the HF + Berry group also had significantly lower relative abundances of Firmicutes (32%, *p* < 0.001), Desulfobacterota (18%, *p* < 0.001), and Actinobacteriota (1%, *p* < 0.05) and a significantly higher relative abundance of Bacteroidetes (12%, *p* < 0.001) (Figure 8).

**FIGURE 9:**
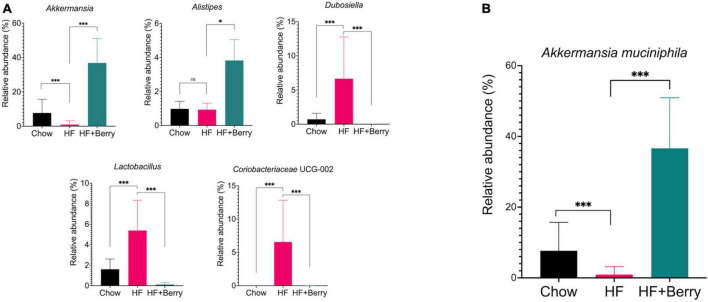
Composition of the gut microbiota at genus and species level in cecum of mice fed chow, HF or HF + Berry diets for 18 weeks. **(A)** Depicts the composition of the gut microbiota at genus level. **(B)** Relative abundance at species level of *Akkermanisa muciniphila*. Values represent mean ± SD for *n* = 12 per diet group. Each group were compared to HF control group using two-way ANOVA and the *p*-values were corrected for multiple comparisons by controlling the False Discovery Rate (FDR) using the original FDR method of Benjamini and Hochberg. Significant differences denoted *p < 0.05 and ***p < 0.001.

At the genus level, the mice in the HF group had significantly higher levels of Dubosiella, Lactobacillus, and Coriobacteriaceae UCG-002 compared to the chow group (*p* < 0.001), as shown in Figure 9A. The mice in the HF + Berry group had significantly higher levels of Alistipes (*p* < 0.05) and lower levels of Dubosiella, Lactobacillus, and Coriobacteriaceae UCG-002 than the HF group. Notably, the main genus from the Verrucomicrobia phylum, Akkermansia, was most abundant in the HF + Berry group at 37%, which was significantly higher than the other groups (Figure 9A).

At the species level, the only significant difference identified between the HF– and the HF + Berry groups was the relative abundance of *Akkermansia muciniphila* among the identified species. The mice fed a high-fat diet supplemented with a berry mixture had the highest relative abundance of *A. muciniphila* at 37% (Figure 9B). This was found to be significantly higher than the chow group (8%, *p* < 0.001) and the HF group (1%, *p* < 0.001).

## 4. Discussion

Our results from this study suggest that the tested mixture of Nordic *vaccinium* berries has a beneficial impact on cognitive function and memory. A diet containing a mixture of lingonberries, bilberries, and grape enhances cognitive function in young mice, as demonstrated by improved spatial memory performance in the T-maze and Barnes maze.

Spatial reference memory can be tested using the T-maze (63) in which mice after some time are expected to remember which arm they have not previously explored and thus visit that arm more often of curiosity. In the current study, mice were fed an HF diet with or without berries from a young age and into adulthood (64). Results from all three parameters assessed at the T-maze showed that the group fed a diet including bilberries and lingonberries preferred the unexplored arm, interpreted as improved spatial memory. These findings are consistent with our previous study (39), where bilberry and lingonberry intake (individually) are linked to improved T-maze performance in middle-aged mice. However, even if improvements were observed with berry supplementation, the high-fat diet *per se* did not necessarily induce cognitive dysfunction, as the HF group did not perform worse than either the chow group nor the HF + Berry group.

To further investigate and clarify the behavior pattern of the diet groups in this cognitive domain, additional tests were included in the study. The Barnes maze test is used to assess hippocampal-dependent spatial learning and memory in different mouse models (65) and is based on the animal’s ability to learn the relationship between distal cues in the surrounding environment and a fixed escape location. The results of the Barnes maze test in this study showed that with training, all diet groups did learn and required a shorter time to find the escape box over time. However, in this test, detrimental effects of the HF diet were observed as the HF group took longer than the HF + Berry group to learn and find the spatial location of the escape hole. Interestingly, the mice getting the berry mixture were protected against the HF-induced reduction of cognitive performance and performed at a similar level or even better than the control group fed the standard chow diet. This indicates that lingonberries, bilberries, and grape mixture can potentially improve hippocampal-dependent spatial learning and memory, in line with the T-maze results in this study as well as a previous study with middle-aged mice (39). To our knowledge, this is the first mouse study testing and showing cognitive outcomes of Nordic berries or even other *Vaccinium* species using the Barnes maze test. However, a limitation is that no impaired performance was observed in the HF group in the probe trial (as was also the case for the chow and the group getting HF with berries), indicating that the mice fed the HF diet were not completely or severely impaired in spatial learning and memory. It is possible that the young age of the mice was protective to some extent against HF-induced behavioral impairment, which also must be counted as a relatively mild model reflecting the lifestyle, rather than genetic or drug-induced cognitive dysfunction (66). It is also important to note that the lack of stressful stimuli, especially during the Barnes maze test, or age-associated anxiety-like behavior can also result in slow learning, which is not necessarily associated with impaired memory (66–68).

No differences amongst the diet groups were noted in the novel object recognition (NOR) test, which assesses recognition memory and preference for novelty in rodents (69). The NOR result indicated that all diet groups explored the novel object more than the familiar object, and no further improvement was obtained by berry supplementation. Nordic berries species may be less beneficial in cognition aspects measured by the NOR test (recognition memory). It is also possible that the result reflects methodological issues or is age-dependent. A previous study has shown that American highbush blueberries (*Vaccinium ashei*) improve HF-induced impairment of recognition memory function in C57Bl/6 male mice fed high-fat diets (20). However, in that particular study, older mice (13 months old at the time of the test) were used.

The discovered cognitive benefits of a lingonberry, bilberry, and grape mixture are supported by data from previous studies on various berry species (29, 38, 39, 70–72). Furthermore, similar positive effects on cognition have been observed in other *Vaccinium* species and grape varieties (28, 29, 71–73). Interestingly, one clinical trial also reported some positive effects on working memory in older healthy adults after 5 weeks of consuming a beverage containing six fruit and vegetable ingredients, including lingonberries and blueberries (74).

Epidemiological studies have indicated a potential correlation between metabolic syndrome - a combination of cardiovascular risk factors including obesity, insulin resistance as well as dyslipidemia - and an increased risk of impaired cognitive function and cognitive disease development (75–78). Lingonberry intake has been associated with various health-promoting effects, including the prevention of body weight gain and hepatic lipid accumulation, reduction of low-grade inflammation, and reduced plasma levels of SAA and LBP in mice fed a high-fat diet (35, 36). Similarly, bilberries or their extract have been shown to prevent body weight gain, reduce systemic inflammation, obesity-associated hypertension, and hyperglycemia in mice fed a high-fat diet (79). Moreover, clinical studies have also demonstrated the efficacy of bilberry and grape seed extract interventions in improving glucose and cholesterol metabolism and blood pressure, according to a review by Chan and Tomlinson (80).

The current study reveals that the berry mixture supplementation provided some protection against HF-induced weight gain and contributed to a significant reduction of liver mass compared to the HF diet alone. The results suggest a healthier metabolic status, as reduced liver size implies protection against hepatic lipid accumulation. Lipid accumulation in the liver, instead of adipose tissue, has been proposed to contribute to insulin resistance (81, 82). In fact, the study shows a trend in the berry group of reduced insulin levels, although non-significant. Previous studies by us and others (83, 84) have shown that a high dose (20%) of lingonberries and bilberries have shown protection against insulin resistance, body weight gain, body fat accumulation, and cholesterol levels in the same mouse strain fed a 45E% high-fat diet. The results on metabolic syndrome-related parameters have been especially promising with lingonberries, with some more mixed results for bilberries. In the current study, the total berry mixture content of the diet was 20% (w/w), meaning that the individual berries were administered at lower concentrations, which could impact results in relation to previous literature using a higher dose. In addition, obesity-related detrimental effects of the 60%E HF diet used in the current study may be more challenging to counteract compared to a berry supplementation to a 45%E HF background diet.

The study also examined the effects of berries and the different diets on markers of inflammation in both blood and in the hippocampus. The results reveal that compared to standard chow, the mice fed a high-fat diet had significantly higher plasma levels of pro-inflammatory cytokines IL-2 and TNFα, as well as the chemokine KC/GRO, which latter plays a role in regulating immune cell migration and recruitment to sites of inflammation and injury (85). However, the berry mixture supplementation had no significant effect on any inflammatory markers measured in plasma. There was also no significant effect of berry supplementation on plasma levels of the acute phase protein SAA. These findings contrast previous studies where lingonberry and bilberry supplementation reduced plasma SAA and hepatic *Saa* gene expression in a slightly different HF-model design (35, 36). Hence, future studies should further evaluate the indications of tendencies toward improved metabolic status in mice receiving the berry mixture. We conclude that the metabolic effects in the study, in particular on systemic inflammation, do not seem to be main drivers of the observed beneficial effects on cognition, which could be rated to the relative young age of the mice.

In addition to examining systemic inflammation, the study also investigated neuroinflammation, which has previously been suggested to be associated with cognitive function (86, 87). It was found that the consumption of the berry mixture did, to some extent, modulate inflammatory status in the brain, as seen by the increased cytokine IL-10 in the hippocampus. IL-10 can inhibit the production of proinflammatory cytokines that play a role in the immune response (88, 89). In the brain, IL-10 expression has been observed not only in microglia but also in astrocytes, oligodendrocytes, and neurons following injury, as reported previously (90). Previous studies have shown that IL-10 expression in the hippocampus of transgenic mice with Alzheimer’s disease (AD) has been shown to increase neurogenesis and enhance cognition, suggesting a neuroprotective role for IL-10 in this pathological condition (91). However, it is worth mentioning that other studies have reported negative effect of IL-10 in AD models, emphasizing the complex role of cytokines and neuroinflammation in AD pathogenesis (92, 93). In the current study, brain cytokine levels were generally low, indicating that the HF diet in the model did not induce profound neuroinflammation. In any event, modulating cytokines production in the brain seems to be an approach to restore a healthy immune response, as opposed to exacerbating adverse effects. The effect on hippocampal anti-inflammatory IL-10 of the berry mixture could constitute an interesting, novel way of action of Nordic lingon- and bilberries concerning brain and cognitive benefits.

Yet another proposed mechanism of berry brain benefits is the promotion of neurogenesis. According to Carey et al. supplementation with highbush blueberry (*Vaccinium ashei*) powder promotes neuroplasticity, indicated by increased numbers of DCX-positive cells (a marker of neurogenesis) and BDNF in the hippocampus of mice fed a high-fat diet supplemented with berry (20). These two parameters were also assessed in the current study and were not altered by berry intake, suggesting that the mode of action may differ between different species of *Vaccinium* berries, possibly due to their different composition of bioactive compounds. It should be noted that conclusions about neurogenesis and neuroinflammation may vary depending on the specific markers and brain regions investigated, and further studies are warranted to understand how Nordic berries may modulate these parameters.

While no effects on neurogenesis were evident, we further explored the proxy of neuronal functionality in the hippocampus by analyzing synaptic proteins. Previous studies suggest significant roles of synaptophysin and GluA1 in maintaining or increasing synaptic strength (94, 95), while pGluA1 at specific sites can regulate the trafficking, insertion, and removal of AMPA receptors from the synaptic membrane, which in turn modulate synaptic strength and plasticity (94, 96–98). In this study, despite no significant difference in GluA1 levels between the HF and HF + Berry groups, the level of pGluA1 was significantly higher in the HF + Berry group, suggesting that the berry supplementation may have a positive effect on the synaptic activity and function. The study also found that the levels of synaptophysin and GluA1 were higher in the chow group compared to the HF group, which aligns with previous studies suggesting that high-fat diet feeding can induce synaptic plasticity and have a detrimental effect on synaptic efficiency (99). A significantly higher level of PSD95 in the HF group compared to the HF + berry seemed to be in contrast with a previous study showing that the consumption of lingonberry supplements significantly increased the total number of synapses and multiple synapses and increased postsynaptic density length in the hippocampus of ApoE–/– mice (100). However, as reported earlier by Martinsson et al. (101), PSD95 protein levels do not always correlate with increased synapse density.

The results in the current study imply that the spatial organization around hippocampal synapses may be disrupted by HF dietary intake. Further research is needed to fully understand the implication of synaptic remodulation for neurological and psychiatric disorders.

Lastly, we studied the gut microbiota composition to identify patterns between specific microbial communities and health outcomes and thus provide valuable information about the potential role the gut-brain axis may play in health.

Interestingly, 20% (w/w) berry intake did not significantly increase the diversity or the total OTU richness of the gut microbiota. These findings were in contrast to our previous study, where a 6% (w/w) of berry supplement to the HF diet showed a significant increase in microbial diversity and richness (39). The higher dose of berry intake may promote the growth of a specific group of bacteria, thus, reducing overall diversity. This is supported by a previous study in which gut microbiota in rats showed increased diversity when administering moderate doses of a purified extract of blueberry polyphenols but decreased diversity at high doses in 5-month-old rats (102).

Moreover, the study results indicate that the diet enriched with the mixture of berries could have a distinctive effect on the gut microbiota composition in mice, as demonstrated by the notable differences in the gut microbial profiles between the HF and the HF + Berry groups. The difference in dietary fiber composition between the HF and HF + Berry diets may have played a role in these differences. Specifically, the dietary fiber in the high-fat group was cellulose only, whereas the high-fat diet supplemented with berries had a more diverse composition of nutritional fibers. Berries are also rich sources of polyphenols (13–16), among these polyphenols, anthocyanins have been shown to be associated with various health benefits in human metabolism (31, 32, 103, 104). One may speculate that these compounds and their metabolites, potentially in synergy with other compounds such as the fiber portion of the berry mixture, contribute to the cognitive and other effects observed in the study. However, as the study design in this work does not allow for a thorough exploration of causal relationships between specific compounds and health outcomes, this subject is important to address in future studies.

Acid hydrolysis showed glucose (48.3%, 1.1) to be the main component of the polymeric material, which is in accordance with a high content of xyloglucans (polymers of β-1,4 linked glucose backbone substituted at O-6 with xylose residues) which has been reported as the main type of hemicellulose in dicotyledonous plants, including bilberries (105). Xylose is most likely also originating from bilberry seeds, where it has been reported to be a significant component (105, 106). The arabinose and galactose are judged to originate from neutral side chains of pectic polymers (e.g., rhamnogalacturonan) reported to contribute up to 0.8 g/100 g of bilberry dry weight (105). Polysaccharides of lingonberries are less investigated but analyses of water-soluble components have shown the main sugars to be arabinose, xylose, galactose, and glucose (107), in line with the current data.

Bacteroidetes are known to be versatile carbohydrate degraders, with enzyme systems that can utilize various types of hemicelluloses, including xyloglucans, as well as arabinoxylans, arabinans and substituted pectin, as identified in the current study. Lingonberries and bilberries were associated with increased relative abundance of Bacteroidetes in our previous work (38, 39). Other studies have also found that many species in Bacteroidetes are linked to help regulate energy metabolism and maintenance of gut barrier function (103, 108, 109). These findings may explain the potential benefits exerted by the berry mixture in terms of weight management control and improved fasting insulin profile in the current study.

An additional finding in this study, *Akkermansia muciniphila* was found to make up about 8% of the microbial community in the cecum of mice fed on the standard chow diet. However, this percentage decreased to 1% in mice fed the high-fat diet. Strikingly, the relative abundance of *A. muciniphila* increased to 37% in mice fed the HF + Berry diet containing bilberries and grape juice, both known for their high levels of anthocyanins and proanthocyanins. These compounds have been associated with promoting the abundance of *A. muciniphila* (104, 110). Several studies from our and other groups have also confirmed the effects of berries and grapes on *Akkermansia* (38, 39, 104, 111–116). Moreover, analysis of available genomes shows that *A. muciniphila* harbors multiple genes classified to encode putative carbohydrate degrading enzymes,^1^ in families with activity for xyloglucan degradation (GH16), as well as for substituent removal of arabinofuranosides (GH43) and galactosides (e.g., GH2 and GH35 and GH110), making it a suitable species for degradation of polysaccharides from berries.

The gut microbiota is shown to be associated with several aspects of human health, even influence the host’s behavior and mood through the so-called “gut–brain axis” (117, 118). The gut microbiota–brain axis refers to the biological systems allowing bidirectional interaction between gut microbes and the brain (117). This interaction involves various pathways within the chemical, neuronal, and immunological systems, as well as the enteric nervous system and the vagnus nerve, the neuroendocrine system, and the hypothalamic-pituitary-adrenal axis (117, 118). *A. muciniphila*, for example, has been implicated in various aspects related to beneficial effects, including reducing adiposity (119, 120), improving insulin resistance, and reducing inflammation (121–123), and alleviation of the neurodegenerative processes (51, 124, 125). One of the mechanisms by which *A. muciniphila* exerts beneficial effects on the host’s gut-brain axis involves direct signaling of outer membrane proteins, metabolite production, and the enhancement of beneficial mucosal microbial networks (126, 127). Interestingly, two clinical studies from the same group reported that a larger proportion of Verrucomicrobia significantly correlates with better performances on cognitive tests (128, 129).

In this study, we found improvement in the cognitive performance on the Barnes maze in the mice fed HF + Berry group. However, whether the beneficial cognitive effect could be linked to the drastic increase in cecal *Akkermansia* needs further studies. On the other hand, the fiber and polyphenol composition of different berries may have varying effects on gut microbiota and other health aspects, impacting brain function. Incorporating a variety of berries into the diet may be an effective way to promote healthy aging and reduce the risk of cognitive decline.

## 5. Conclusion

The findings in this study demonstrate that a Nordic berry mixture improves spatial memory performance in mice fed on a high-fat diet. This beneficial cognitive effect may relate to an observed modulation of neuroinflammation and synaptic function in the hippocampus, a brain region essential for spatial learning and memory.

In addition, the berry mixture was found to affect whole-body metabolism, which may also be linked to improved brain function, for example, by providing some protection against weight gain caused by a high-fat diet as well as promoting a shift in gut microbiota composition, notably by an increased relative abundance of the bacterium *Akkermansia muciniphila*. Overall, this study adds to the growing body of research on the potential benefits of berries for brain health, metabolic function, and gut microbiota research and establishes that Nordic berries are exciting candidates for dietary strategies to prevent disease. Further research and clinical trials are ongoing and required to fully understand the potential cognitive benefits of more varieties of berries or fruit species.

## Data availability statement

The 16S RNA sequence datasets have been deposited in the publicly accessible Sequence Read Archive (SRA) repository, under the accession number PRJNA1013755. Please refer to the following link: https://www.ncbi.nlm.nih.gov/bioproject/PRJNA1013755.

## Ethics statement

The animal study was approved by the local animal experiment Ethical Review Committee in Lund, Sweden (approval number 5.8.18–13,983/2018). The study was conducted in accordance with the local legislation and institutional requirements.

## Author contributions

FH: Conceptualization, Methodology, Data acquisition, Data curation, Formal analysis, Investigation, Data visualization, Writing – original draft preparation, Writing – review and editing. NM: Conceptualization, Methodology, Data acquisition, Formal analysis, Investigation, Data visualization, Writing – review and editing. IM: Data acquisition, Data curation, Formal analysis, Investigation, Data visualization, Writing – review and editing. LCF: Methodology, Data acquisition, Data curation, Writing – review and editing. TN: Data acquisition. TG: Methodology, Data acquisition, Formal analysis, Writing – review and editing. EK: Writing – review and editing, Supervision. TD: Writing – review and editing, Supervision. RÖ: Supervision. LH-L: Conceptualization, Methodology, Investigation, Writing – review and editing, Supervision.

## Footnotes

1
www.cazy.com
