# Supplementary material for: A mixture of Nordic berries improves cognitive function, metabolic function and alters the gut microbiota in C57Bl/6J male mice
Source: Front Nutr. 2023 Oct 3;10:1257472. doi: 10.3389/fnut.2023.1257472 (PMC10580983; doi:10.3389/fnut.2023.1257472)
Supplement: Supplementary file 1 [file Data_Sheet_1.docx]

**Supplement Materials**

| Table S1  Composition of rodent diets used in the study. All diets were designed to have an equal caloric content from fat, protein and carbohydrates. | | | | | |
| --- | --- | --- | --- | --- | --- |
|  | HF control^5^ | | HF+Berry |  |  |
| *Calculated energy(kcal)* |  | |  |  |  |
| Protein | 708 | | 708 |  |  |
| Carbohydrate | 815 | | 815 |  |  |
| Sucrose | 315 | | 5 |  |  |
| Fructose | 0 | | 152 |  |  |
| Glucose | 0 | | 147 |  |  |
| Fat | 2430 | | 2430 |  |  |
| Fiber | 0 | | 0 |  |  |
| Other | 0 | | 0 |  |  |
| Total kcals | 3953 | | 3953 |  |  |
| *Calculated energy per gram diet (kcal/g)* | | |  |  |  |
| *kcal/g* | | 5.1 | 5 |  |  |
| *Calculated energy (kcal%)* | |  |  |  |  |
| Protein^1^ | | 18 | 18 |  |  |
| Carbohydrate^2^ | | 21 | 21 |  |  |
| Fat^3^ | | 61 | 61 |  |  |
| Fiber^4^ | | 0 | 0 |  |  |
| Fiber content^3^(g/100g diet) | | 6.5 | 6.3 |  |  |
| ^1^ For both diets, protein consisted primarily from added casein (comprising more than 98 % by weight) and L-Cystine.  ^2^ In the HF control diet, carbohydrates were provided through added sucrose (34% by weight), maltodextrin (61% by weight), and a mineral mix (5% by weight), which also facilitated the rodent pellet formulation and was considered as part of the carbohydrate content. The HF+Berry diet's carbohydrate composition primarily included simple sugars (such as glucose and fructose) sourced from the berry powder, along with added sugars, maltodextrin (47% by weight), and the same mineral mix (5% by weight).  ^3^ The fat content in the HF control diet consisted of 91% lard and 9% soybean oil by weight. Meanwhile, the HF+Berry diet included 91% lard and 7% soybean oil, with the remaining proportion coming from the fat present in the berries.  ^4^Fiber coming from added cellulose (HF control diet) or added cellulose plus fiber present in berries (HF+berry diet) formulated to obtain diets matched on total fiber content.  ^5^The HF control diet is based on a slightly modified D12492 diet, with the ingredients listed here: <https://researchdiets.com/formulas/d12492> | | | | | |

| Table S2  Nutritional composition of the freeze-dried berry powder | | |
| --- | --- | --- |
| Macronutrients of the berry powder | | |
| g/100g | Mean | SD |
| fat | 3.50 | 0.18 |
| Carbohydrates | 68.0 | 4.80 |
| Glucose | 23.2 | 4.60 |
| Fructose | 24.0 | 4.80 |
| Galactose | 0.19 | 0.04 |
| others | 20.6 |  |
| Fiber | 13.7 | 2.70 |
| Protein | 3.95 | 0.20 |
| Water content | 9.20 | 0.09 |
| Composition of the carbohydrates of the berry powder | | |
| g/100g | Mean | SD |
| Arabinose | 1.80 | 0.1 |
| Galactose | 1.20 | 0.1 |
| Glucose (hydrolysed) | 48.3 | 1.1 |
| Free glucose | 23.2 | 4.6 |
| Other glucose | 25.1 |  |
| Free fructose | 24.0 | 4.8 |
| Xylose | 1.90 | 0.1 |
| Total phenolics, total flavonoids, and total anthocyanins of the berry powder (n=3) | | |
| mg/g | Mean | SD |
| Total phenolics | 8.77 | 0.24 |
| Total flavonoids | 1.46 | 0.04 |
| Total anthocyanins | 8.61 | 0.20 |

Figure S1. Diet influences levels of synapse related proteins in hippocampal homogenates. (a) GluA1; (b) pGluA1 Ser 845; (c) FOSB; (d) CREB; (e) Synaptophysin; (f) PSD95; (g) pCREB ser 133; (h) NR1. One-way ANOVA followed by Dunnett’s test was applied for multiple comparisons versus the HF control group. Significant differences denoted * p <0.05, *** p < 0.001. Values are represented as mean ± SD for n = 10 –12 per diet group. Images of the Western blot are shown, displaying representative samples for each protein of interest. These images are indicative of three independent experiments, with the respective control bands for tubulin provided below each corresponding protein.

Figure S2. Blood plasma level of LBP (ng/ml) in the chow–, the HF– and the HF+Berry group. One-way ANOVA followed by Dunnett’s test was applied for multiple comparisons versus the HF control group. Significant differences denoted *** p < 0.001. Values are represented as mean ± SD for n = 11–12 per diet group.

Figure S3. Blood plasma levels of (a) Total cholesterol (mmol); (b) HDL (mmol) in the chow, HF– and the HF+Berry group. One-way ANOVA followed by Dunnett’s test was applied for multiple comparisons versus the HF control group. Significant differences denoted *** p < 0.001. Values are represented as mean ± SD for n = 12 per diet group.


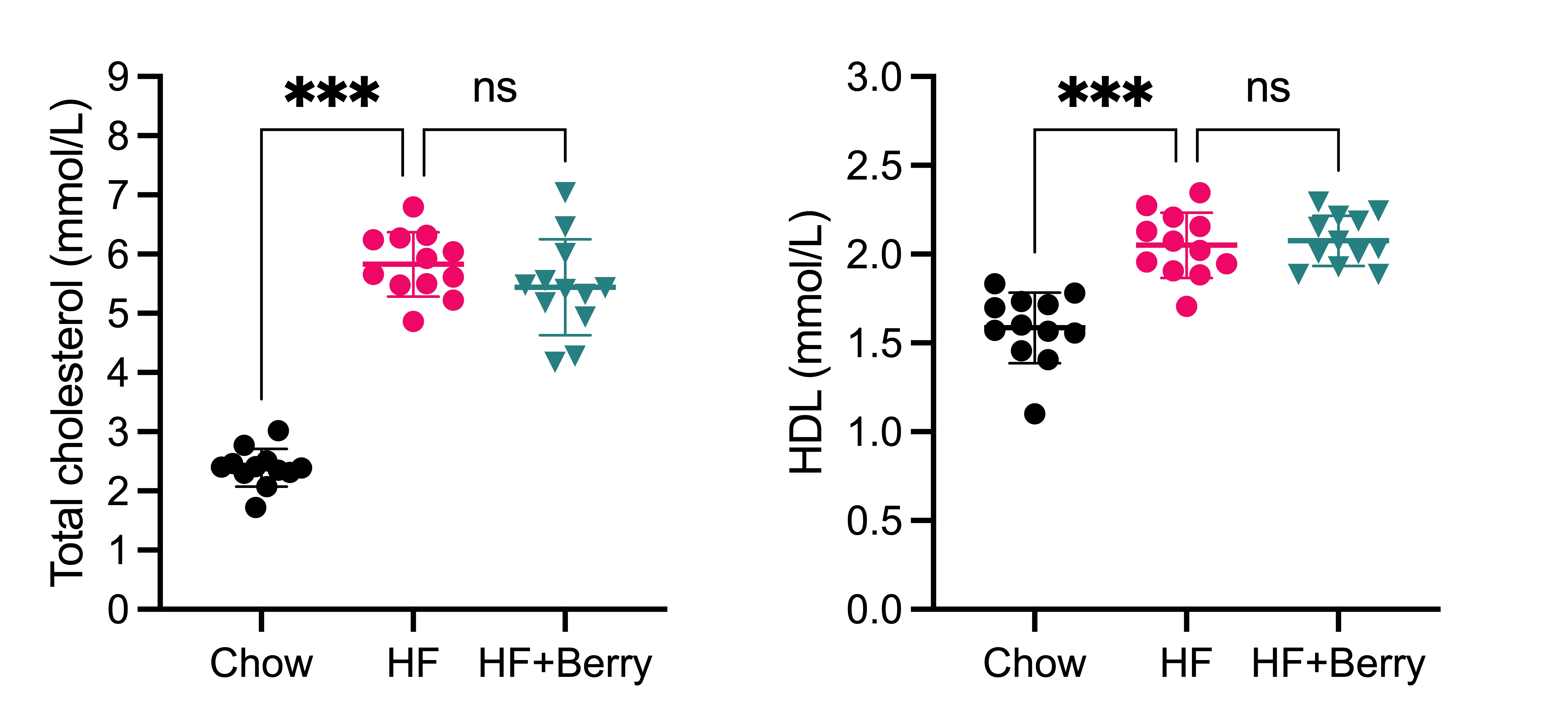


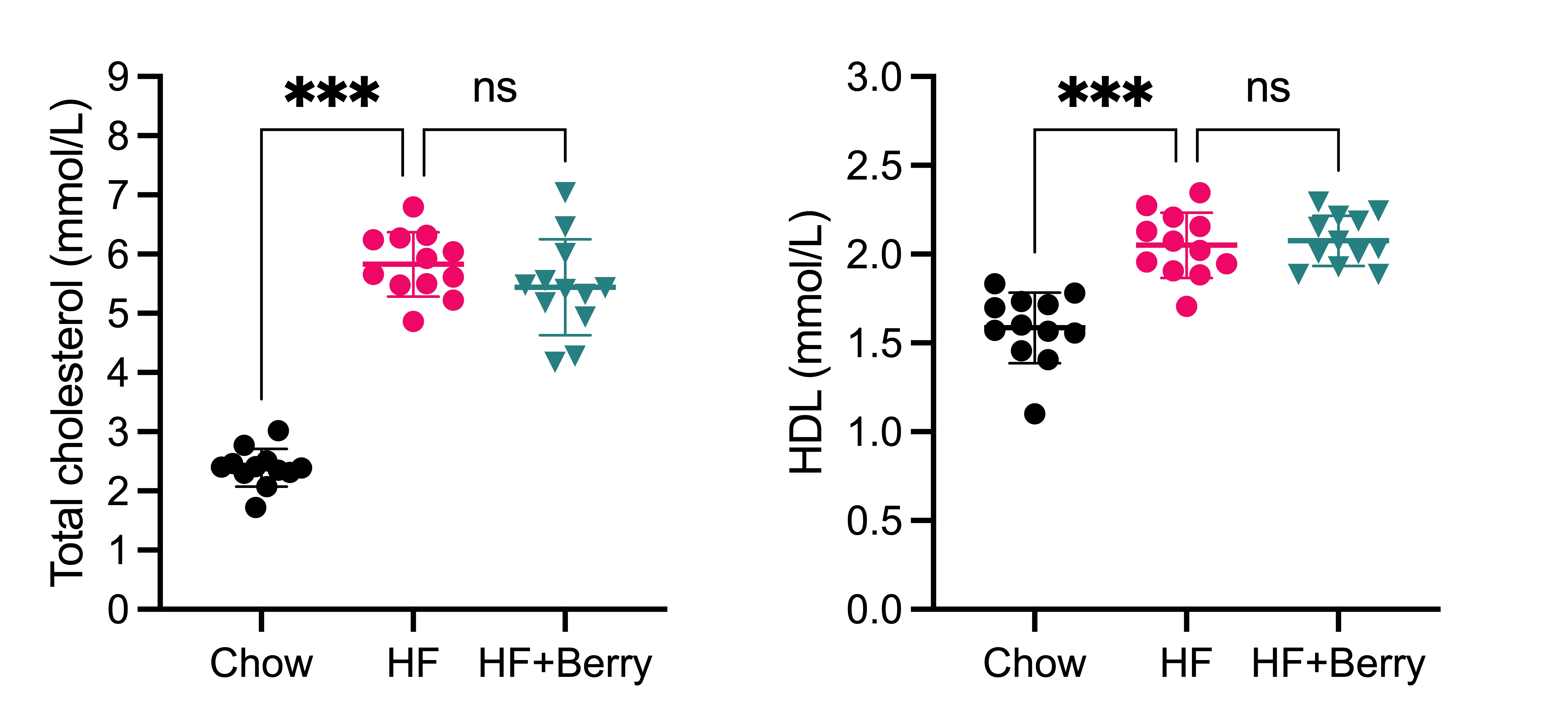

Figure S4. The effect of chow, HF and HF+Berry diets on cytokine levels in the plasma. Mesoscale results are given in pg/ml for all cytokines. (a)IFNγ; (b)IL-10; (c)IL-2; (d)IL-5; (e)IL-6; (f)KC/GRO; (g)TNFα. One-way ANOVA followed by Dunnett’s test was applied for multiple comparisons versus the HF control group. Significant differences denoted *** p < 0.001. Values are represented as mean ± SD for n = 10–12 per diet group.
